# The Uricosuric Effect of SGLT2 Inhibitors Is Maintained in the Long Term in Patients with Chronic Kidney Disease and Type 2 Diabetes Mellitus

**DOI:** 10.3390/jcm13051360

**Published:** 2024-02-27

**Authors:** Paula Sánchez-Briales, María Marques Vidas, Paula López-Sánchez, María Victoria López-Illázquez, Lucía Martín-Testillano, Aylin Vedat-Ali, Jose Portolés

**Affiliations:** 1 Nephrology Department, Hospital Universitario Puerta de Hierro Majadahonda, Instituto de Investigación Sanitaria Puerta de Hierro-Segovia de Arana (IDIPHISA), 28222 Madrid, Spain; paula.sanchezbriales@gmail.com (P.S.-B.); nefro_metodologia@yahoo.com (P.L.-S.); nefro.huphm@gmail.com (M.V.L.-I.); lumartin.testillano@gmail.com (L.M.-T.); aylinvedatali7@gmail.com (A.V.-A.); josem.portoles@salud.madrid.org (J.P.); 2 Medicine Department, Facultad de Medicina, Universidad Autónoma de Madrid, 28222 Madrid, Spain

**Keywords:** chronic kidney disease, fractional excretion, SGLT2 inhibitors, type 2 diabetes mellitus, uric acid

## Abstract

(1) Background: Sodium–glucose co-transporter 2 inhibitors (SGLT2is) increase uric acid excretion. The intensity of uricosuria is linked to glycosuria. (2) Methods: We aim to analyze the effect of SGLT2 inhibitors on urinary fractional excretion (FE) of uric acid and glucose in patients with type 2 diabetes mellitus (T2DM) and chronic kidney disease (CKD) in a single-center retrospective study with patients with T2DM and CKD who started on treatment with SGLT2is. Patients on renal replacement therapy or with glucagon-like peptide-1 (GLP1) analogs were excluded. Subgroup analysis was performed according to the estimated glomerular filtration rate (eGFR), the SGLT2i molecule, the main comorbidities, and concomitant treatment. As a secondary objective, the study analyzed the effect of SGLT2 inhibitors on uricemia levels. (3) Results: Seventy-three patients were analyzed, with a mean follow-up of 1.2 years. Uric acid and glucose FE significantly increased after the initiation of SGLT2is. This increase remained stable during the follow-up without differences among eGFR groups. No significant reduction in uricemia was observed. However, a trend towards a decrease was observed. (4) Conclusion: The use of SGLT2is in patients with CKD and T2DM is associated with an increase in uric acid FE, which maintains stability irrespective of glomerular filtration loss at least during 24 months of follow-up.

## 1. Introduction

Hyperuricemia is associated with a higher risk of cardiovascular events, hypertension, and the development and progression of chronic kidney disease (CKD). However, the efficacy of xanthine oxidase inhibitors, including febuxostat, as cardio- and nephroprotective agents remains unclear in intervention studies [1].

Sodium–glucose co-transporter 2 (SGLT2) inhibitors have shown extensive benefits that extend beyond glycemic control, including their ability to reduce serum uric acid, and are particularly notable for their cardioprotective and renoprotective effects [2]. SGLT2 inhibitors have proven effective and safe in the management of heart failure. The cardioprotective effects of SGLT2 inhibition involve multiple mechanisms, including reducing preload and afterload, enhancing cardiac metabolism, inhibiting myocardial Na^+^/H^+^ exchange, and reducing cardiac fibrosis and necrosis. Additionally, they exhibit renoprotective effects by restoring tubuloglomerular feedback and reducing renal inflammation and fibrosis. Clinical studies provide evidence supporting their use in mitigating the risk of kidney disease progression and acute kidney injury [3].

The uric acid levels in the blood depend on a delicate balance between hepatic production and elimination. In healthy individuals, roughly one-third of uric acid is eliminated through the intestines, while the remaining two-thirds is excreted via the kidneys. The renal fractional excretion (FE) of uric acid is approximately 10%, which results from complex mechanisms of filtration, reabsorption, and tubular secretion [4]. These mechanisms involve several transporters responsible for uric acid reabsorption, predominantly found in the proximal convoluted tubule of the human kidney. Certain drugs, like benzobromarone, probenecid, losartan, and acetylsalicylic acid (ASA), inhibit these transporters, resulting in a uricosuric effect, while others, such as insulin, promote uric acid reabsorption [4].

SGLT2 inhibitors play a pivotal role in the reabsorption of approximately 90% of the glucose filtered by the kidney, with the remaining 10% being reabsorbed by sodium–glucose co-transporter 1 [4].

SGLT2 inhibitors have demonstrated hypouricemic effects in both patients with and without type 2 diabetes mellitus (T2DM) [5,6,7,8,9]. This hypouricemic effect is primarily attributed to an increase in the FE of uric acid [4,10]. Notably, the hypouricemic effect is not significant in patients with an estimated glomerular filtration rate (eGFR) below 60 mL/min/1.73 m^2^ [7,8]. However, it is important to note that no studies have been specifically designed for this population. The mechanism by which SGLT2 inhibitors enhance uric acid elimination is not fully understood. It has been observed that this effect is not a direct action of the drug (as SGLT2 does not transport urate), but rather a consequence of increased glucose levels in the renal tubules [4,11]. In addition to the tubular effects of SGLT2 inhibitors, it is believed that the reduction in uric acid levels may also be influenced by weight loss and improved insulin resistance [9].

This study aims to investigate whether SGLT2 inhibitors increase the FE of uric acid in patients with CKD and T2DM and whether this effect remains constant over 12- and 24-month intervals.

## 2. Materials and Methods

A retrospective single-center observational study (January 2018 to December 2022) was conducted on patients with T2DM and CKD attended in our dedicated outpatient clinic and with a new SGTL2 inhibitor prescription. Patients on renal replacement therapy (RRT), patients receiving glucagon-like peptide-1 (GLP-1) analog treatment, and patients without baseline uricosuria data were excluded. 

The study’s primary objective was to analyze whether the FE of uric acid and glucose changed over time or remained stable. 

As a secondary objective, the study analyzed whether SGLT2 inhibitors reduced uricemia levels or decreased the dose of uric acid-lowering medications.

Data were collected at baseline and up to 24 months of follow-up, with cut-off points at 3, 6, and 12 months. The formula for calculating FE was as follows: FE = 100 × (urine uric acid) × (serum creatinine)/(serum uric acid) × (urine creatinine). First-morning urine was used, as it has demonstrated a good correlation with 24 h urine samples regarding FE [12]. 

Subgroup analysis of uric acid FE was performed based on significant comorbidities: arterial hypertension (HBP), obesity, and congestive heart failure (HF) and also with various drugs involved in uric acid metabolism that could act as confounding factors (insulin, ASA, thiazides, loop diuretics, losartan, allopurinol, and febuxostat). Additionally, uric acid FE was analyzed based on the SGLT2 inhibitor molecule. 

Additionally, analysis of uric acid FE was performed in the different CKD stages according to eGFR: stages 1 and 2 (eGFR > 60 mL/min/1.73 m^2^, group 1), stage 3a (eGFR 45–59 mL/min/1.73 m^2^, group 2), stage 3b (eGFR 30–44 mL/min/1.73 m^2^, group 3), and stage 4 (eGFR < 30 mL/min/1.73 m^2^, group 4). eGFR was calculated using the CKD-EPI formula [13]. 

Statistical analysis: The sample size necessary to evaluate the main result was estimated, considering a minimum difference of 1.5 and a standard deviation of 4.0. Continuous variables were presented as means and standard deviations (SDs) or median and interquartile range (IQR), and categorical variables as valid percentages. Comparisons between groups were performed using the Chi-Square or Fisher test for qualitative variables and the t-Student paired test/ANOVA repeated samples for quantitative variables. A nonparametric trend test was performed to evaluate trends of uricemia. The Kolmogorov–Smirnov test was used to determine the normality of the distribution of the data.

A *p*-value < 0.05 was considered statistically significant. The statistical package STATA 14.0 (Stata Statistical Software: Release 14. College Station, TX, USA: Stata Corp LP) was used for the statistical analysis.

Data were anonymized, ensuring the confidentiality of the patients. This study was approved by the Ethics Committee of the Hospital Universitario Puerta de Hierro (Number 163/22).

## 3. Results

**Figure 1:**
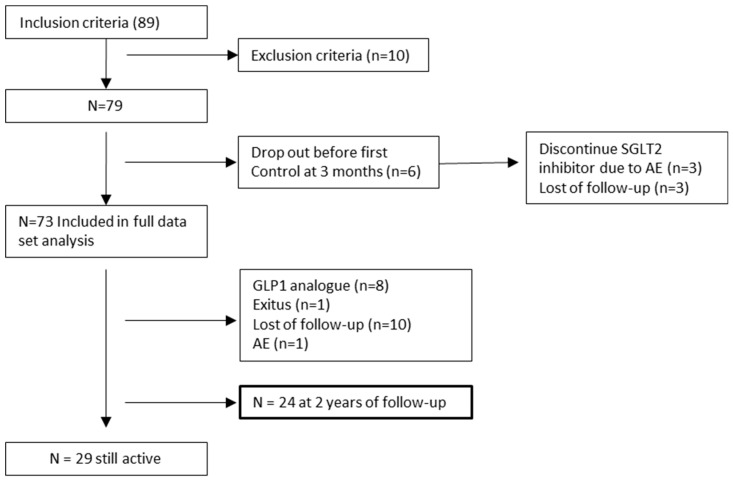
Patient flow chart. Note: *n* = number of patients, SGLT2: sodium–glucose co-transporter 2, GLP1: glucagon-like peptide-1, AE: adverse event.

According to the sample size estimated (*n* = 58 plus 20% due to loss of follow-up), 89 patients were included in the study, of which 73 could finally enter the analysis (Figure 1), with a mean follow-up of 1.2 (SD 0.7) years. During the study, some patients ceased to attend the clinic, leading to a loss of follow-up (three before the initial analysis and ten throughout the follow-up period, Figure 1). In certain instances, follow-up was interrupted during the COVID-19 pandemic, leading to patients not resuming their clinic visits. Additionally, one patient developed genital candidiasis after three months of treatment, resulting in the discontinuation of the SGLT2 inhibitor.

Baseline characteristics and primary comorbidities are shown in Table 1. The distribution of patients according to baseline eGFR was stage 1, 11 patients (15.1%); stage 2, 32 patients (43.8%); stage 3, 29 patients (39.7%); and stage 4 (1.4%).

The median dosages of different SGLT2 inhibitor molecules were as follows: canagliflozin, 100 mg/day; dapagliflozin and empagliflozin, 10 mg/day. The dosage remained stable throughout the two years of follow-up. A single patient developed genital candidiasis after three months of treatment, and the SGLT2 inhibitor was discontinued. 

**Figure 2:**
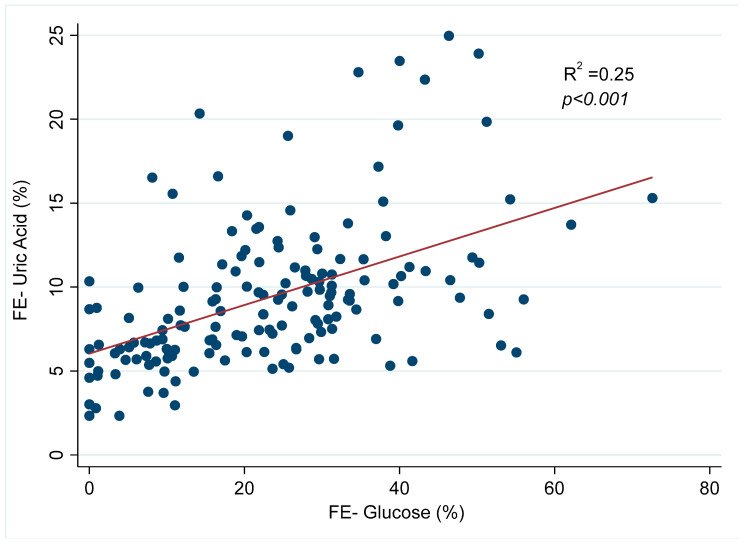
Relationship between uric acid and glucose fractional excretion. Note. FE: fractional excretion.

We observed that uric acid FE followed a distribution directly proportional to glucose FE (Figure 2).

Uric acid and glucose FE significantly increased after the initiation of SGLT2 inhibitor treatment. This increase remained stable during the follow-up period and showed no differences among eGFR groups (see Table 2 and Table 3). 

Serum uric acid levels did not significantly decrease after initiating SGLT2 inhibitors (repeated measures ANOVA, *p* = 0.17), and there was no trend of decrease (*p* = 0.3). However, a trend towards a decrease in uricemia remained stable throughout the follow-up period (Table 2). 

Nineteen patients were receiving allopurinol treatment, and six received febuxostat at baseline. Allopurinol was initiated at the same time as the SGLT2 inhibitor in a single patient. The allopurinol and febuxostat doses remained unchanged in all patients, except for one patient whose allopurinol treatment was discontinued after six months and another one in whom febuxostat was discontinued after three months. No hypouricemic treatment was initiated in any patient following the initiation of SGLT2 inhibitors. 

**Figure 3:**
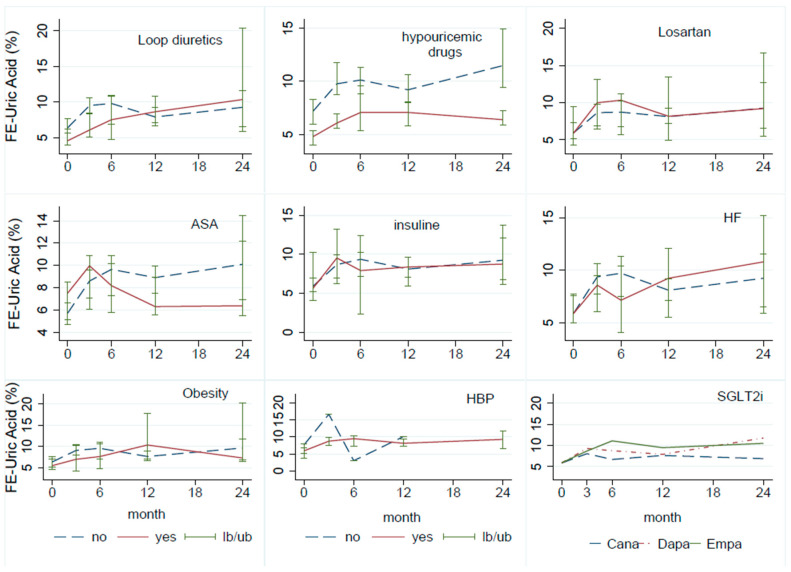
Fractional excretion (FE) of uric acid in subgroup analysis by drugs used and comorbidities. Note: FE: fractional excretion, ASA: acetylsalicylic acid, HF: heart failure, HBP: high blood pressure, SGLT2i: sodium–glucose co-transporter 2 inhibitor.

Subgroup analysis was conducted for the uric acid FE (Figure 3). The uric acid FE was similar for all three SGLT2 inhibitor molecules. A lower uric acid FE was observed in patients receiving uric acid-lowering medications and ASA treatment, although this difference was not statistically significant. No differences were observed in the uric acid FE for other concomitant medications and comorbidities. 

## 4. Discussion

Our study suggests that SGLT2 inhibitors prescribed in CKD and T2DM patients are associated with increased uric acid fractional excretion (FE), which remains directly proportional to glucose FE over time, irrespective of glomerular filtration loss. These results suggest a direct effect on tubular handling of uric acid directly depending on increased glucose delivery to the proximal tubule.

Hyperuricemia is a cardiovascular risk factor both in the general population [5,14] and in CKD patients [14]. Hyperuricemia is associated with kidney damage, with several proposed mechanisms including increased oxidative stress, endothelial dysfunction, glomerular hypertension, induction of fibrosis and glomerulosclerosis, and tubular damage due to urate crystal deposition, among others [15]. Small clinical trials have shown an improvement in kidney disease progression, hospitalization rates, and cardiovascular risk in patients treated with uric acid-lowering agents [1]. However, other studies [16,17,18] have failed to demonstrate these benefits. Moreover, it has been observed that the relationship between serum levels of uric acid and mortality in patients with chronic kidney disease (CKD) follows a U-shaped pattern. UA levels above 10 mg/dL indicate a significant risk of renal failure and death, while lower UA levels below 5 mg/dL are associated with a higher risk of death before renal failure occurs [19].

Due to the controversy in the results of the intervention studies, current guidelines do not recommend the treatment of asymptomatic hyperuricemia in patients with CKD and/or high cardiovascular risk [20].

Nonetheless, it has been suggested that one of the mechanisms underlying the nephroprotective effect of SGLT2 inhibitors is their ability to induce uricosuric effects. Several studies [5,6,7,8] have shown that SGLT2 inhibitors decrease serum uric acid levels in both diabetic and non-diabetic populations. This hypouricemic effect is due to an increased uric acid FE [4,10]. In a meta-analysis, it became evident that the extent of the hypouricemic effect varied depending on the specific SGLT2 inhibitor used, with canagliflozin demonstrating a more pronounced effect compared to empagliflozin and dapagliflozin [8]. However, contradictory findings were reported in other meta-analyses [6]. Some studies indicated that the hypouricemic effect was dose-dependent for SGLT2 inhibitors [6,9], while others found that the effect was not influenced by the dosage [7,8].

Across most studies, the average reduction in uric acid levels was approximately 0.6 mg/dL [5,6], suggesting that these drugs may not be particularly potent in this regard. Nevertheless, they could play a complementary role in the treatment of hyperuricemia.

To the best of our knowledge, no previous study has explored the measurement of uric acid FE in patients with CKD. Our study, however, revealed a sustained increase in uric acid FE in individuals with both CKD and T2DM who were treated with SGLT2 inhibitors over 12- and 24-month periods. This observation implies that there may be no counterbalancing mechanisms in place within the transporters responsible for uric acid handling, or at the very least, these mechanisms are insufficient to mitigate the long-term uric and glycosuric effects.

As reported by others [4,10], the increase in uric acid FE was strongly linked to an increase in glucose FE. This elevated presence of glucose in the renal tubules competes with uric acid reabsorption, primarily through various transporters, with GLUT9 being the most significant, leading to a uricosuric effect [4]. Our study’s increase in uric acid FE closely resembled what was observed in patients with normal renal function and T2DM [10]. Furthermore, our investigation demonstrates that uric acid and glucose FE values remain similar regardless of the eGFR. These findings suggest that the tubular transporters involved appear to be unaffected by the progression of CKD. Another conceivable explanation involves the adaptive upregulation of glucose co-transporters as a compensatory response to sustained hyperglycemia. This adjustment, alongside the complex interplay of various regulatory pathways that govern glucose transport, could be pivotal in preserving the functionality of glucose co-transporters even as the disease progresses. This hypothesis underscores the necessity for further investigative efforts to elucidate the precise underlying mechanisms responsible for this observed adaptation [21].

However, while a trend towards hypouricemia was observed, we did not achieve statistical significance in reducing plasma uric acid levels or administering medications to lower uric acid. 

The absence of statistical significance may be attributed to limited statistical power. Likewise, in other studies, this hypouricemic effect was not evident in patients with an eGFR below 60 mL/min/1.73 m^2^ [6,8], even though these investigations were not specifically tailored for this subgroup. It is also conceivable that with a reduced eGFR, the overall elimination of uric acid becomes less effective, even if FE remains consistent. A potential explanation for the observed discrepancy between the increased uric acid FE and the stable levels of uric acid could involve compensatory renal mechanisms. Specifically, the kidney’s proximal tubule may enhance uric acid reabsorption to maintain the systemic equilibrium of this metabolite. Furthermore, the use of SGLT2 inhibitors is associated with alterations in renal hemodynamics, which could affect the handling of uric acid by the kidneys. This impact may be mediated through intricate interactions among glomerular filtration and tubular reabsorption and secretion processes, thereby influencing the regulation of uric acid levels [10].

Notably, Chino et al. [7] observed that a more substantial initial uric acid level was associated with a more pronounced hypouricemic effect. Therefore, it is plausible that the lack of significant differences in the hypouricemic effect in our study may be attributed to the relatively low mean uric acid levels (6.6 mg/dL).

No discernible differences were observed in the predefined subgroup analyses. However, the limited sample size in some subgroups may explain the lack of differentiation. While certain trends are apparent, these findings should be interpreted cautiously. In our study, there were no significant disparities in uric acid FE based on the specific SGLT2 inhibitor used, indicating a consistent class effect, in line with findings from other studies [6].

Additionally, there were no noticeable differences among patients with HBP, obesity, or HF. However, we did observe a reduction in the FE of uric acid in individuals receiving treatment for lowering uric acid levels. This observation could be explained by the fact that FE may decrease as plasma uric acid levels decrease [7].

An unexpected observation was a diminished uric acid FE in patients receiving ASA treatment, even though ASA is recognized for its uricosuric effect, leading us to expect the opposite. This finding lacks statistical significance and should be cautiously approached due to the small sample size of individuals using this drug. Nevertheless, it might warrant further investigation through specifically designed studies.

This study has several limitations: (1) it is a retrospective, single-center study; (2) the study group is heterogeneous regarding comorbidities, renal function stage, and concomitant medication; (3) some CKD stages are poorly represented; and (4) this study was conducted on patients with T2DM and CKD. It is unknown whether SGLT2 inhibitors will have the same effect on the uric acid FE in non-T2DM CKD patients since diabetes modifies uric acid elimination.

The study has notable strengths, including the use of subgroup analyses to address the limitations arising from the heterogeneity of the patient group, which enhances the external validity of the results. Moreover, it is worth noting that patient series with routine assessments of both glucose and uric acid in urine are exceptionally rare, giving the findings of this study unique value, even in light of their retrospective nature.

## 5. Conclusions

In summary, SGLT2 inhibitors increase uric acid FE in the CKD and T2DM population. This increase is directly linked to glucose FE and is maintained over time, regardless of eGFR loss. This may have implications for understanding the mechanism of SGLT2 inhibitors in this patient population. 

## Figures and Tables

**Table 1:** Baseline characteristics, comorbidities, and concomitant drugs with an effect on uric acid metabolism.

Patients (*n*)	73
Male (%)	78.1
Age (y; mean, SD)	72.2 (8.7)
Caucasian (%)	95.0
Comorbidities (%)	
*High blood pressure*	95.9
*Hyperuricemia*	74.0
*Heart failure*	26.0
*Obesity*	18.5
*Dyslipidemia*	68.4
CKD etiology (%)	
*Diabetes mellitus type 2*	49.3
*Nephroangiosclerosis*	16.0
*Glomerulonephritis*	4.1
*Interstitial*	9.5
*Others*	20.4
Drugs involved in uric acid metabolism (%)	
*Losartan*	16.4
*Insulin*	19.1
*Loop diuretics*	27.4
*Tiazide*	38.4
*Acetylsalicylic acid*	27.4
*Alopurinol*	26.0
*Febuxostat*	8.2
SGLT2 inhibitor drugs (%)	
*Canagliflozina*	25.8
*Dapagliflozina*	61.3
*Empagliflozina*	9.7

Y: years; SD: standard deviation; CKD: chronic kidney disease; SGLT2: sodium–glucose co-transporter 2.

**Table 2:** Uric acid and glucose fractional excretion.

Time	Baseline	Month 3	Month 6	Month 12	Month 24
N	73	61	45	45	24
Uric acid FE (%)	5.9 [4.4–8.3]	8.7 [6.1–11.2] *	9.1 [6.3–10.8] *	8.2 [6.7–10.3] *	9.6 [6.5–12.2] *
Glucose FE (%)	0	20.1 [8.6–30.8] *	25.3 [11.8–31.3] *	21.9 [11.0–32.3] *	24.3 [14.2–38.8] *
eGFR (mL/min/1.73 m^2^)	46 [40.0–55.0]	39 [34.0–52.0] *	37 [33.0–44.0] *	38.5 [32.0–47.5] *	40 [31.0–54.0] *
Serum uric acid (mg/dL)	6.6 [5.6–7.8]	6.4 [5.0–7.5]	6.4 [4.8–7.5]	6.2 [5.2–7.6]	6.2 [5.2–7.0]
Serum glucose (mg/dL)	131 [111.0–146.0]	124 [113.0–141.0]	126 [105.0–152.0]	133 [115.0–155.0]	120 [112.0–136.0]

Laboratory data are expressed as the median and interquartile range [IQR] with paired *t*-test vs. basal values. * *p* value < 0.001. No significant differences between other values. FE: fractional excretion, eGFR: estimated glomerular filtration rate, *n* = number of patients.

**Table 3:** Uric acid and glucose FE follow-up stratified by chronic kidney disease.

	Basal eGFR	Baseline	Month 3	Month 6	Month 12	Month 24
Uric acid FE (%)	30–45 (n30)	5.6 [4.3–7.9]	9.3 [6.4–13.7]	10.0 [6.7–11.4]	7.6 [7.1–11.7]	9.2 [5.9–20.3]
	45–60 (n32)	6.8 [4.8–8.9]	8.6 [6.1–12.2]	8.8 [5.1–9.6]	8.4 [6.5–9.6]	7.8 [6.4–10.9]
	>60 (n11)	5.6 [4.4–5.8]	8.8 [6.3–10.0]	7.7 [6.9–10.3]	8.7 [7.1–11.8]	11.5 [10.2–12.7]
Glucose FE (%)	30–45 (n30)	0 [0.0–0.0]	19.6 [8.1–31.8]	28.1 [12.1–46.5]	18.0 [9.7–32.3]	12.9 [10.6–16.4]
	45–60 (n32)	0 [0.0–0.0]	21.2 [12.6–31.1]	24.6 [16.9–30.0]	26.2 [22.1–33.5]	28.1 [20.2–41.1]
	>60 (n11)	0 [0.0–0.0]	13.2 [7.6–27.9]	15.9 [11.8–29.6]	19.3 [8.5–21.9]	37.0 [24.3–37.3]

Laboratory data are expressed as the median and interquartile range (IQR). eGFR: estimated glomerular filtration rate (mL/min/1.73 m^2^), FE: fractional excretion, *n* = number.

## Data Availability

Data will be available after a formal request to the corresponding author.
